# Aconiti Lateralis Radix Preparata, the Dried Root of *Aconitum carmichaelii* Debx., Improves Benign Prostatic Hyperplasia via Suppressing 5-Alpha Reductase and Inducing Prostate Cell Apoptosis

**DOI:** 10.1155/2019/6369132

**Published:** 2019-07-31

**Authors:** Jinbong Park, Dong-Hyun Youn, Jae-Young Um

**Affiliations:** ^1^Department of Pharmacology, College of Korean Medicine, Kyung Hee University, Seoul, Republic of Korea; ^2^Comorbidity Research Institute, Kyung Hee University, Seoul, Republic of Korea

## Abstract

Benign prostatic hyperplasia (BPH) is a common disease in elderly men which can be characterized by an abnormal enlargement of the prostate associated with lower urinary symptoms. Current medications available for BPH treatment display several adverse effects; thus, the search for effective treatments with less side effects is still ongoing. In this study, we investigated the effect of Aconiti Lateralis Radix Preparata (dried root of *Aconitum carmichaelii* Debx.; AL), which is an herb used to treat extremely cold symptoms in traditional Korean medicine, on BPH using a testosterone propionate- (TP-) induced BPH rat model. Eight-week inguinal injection of TP induced BPH in rats, the prostate of which was displaying an abnormal proliferation. The pathological proliferation of the prostate was ameliorated by AL treatment of 4 weeks. Pathohistological changes in the prostate including epithelial thickness and lumen area were restored in AL-treated rats. Furthermore, 5*α*-reductase (5AR) and androgen receptor (AR), the two main factors in the pathogenesis of BPH, were decreased. In addition, the ratio of BAX and Bcl-2, an indicator of apoptosis, was increased by AL as well. Similar results were observed in AL-treated LNCaP prostate cancer cells. AL treatment suppressed the expression of the 5AR-AR axis and increased the ratio of BAX and Bcl-2. Apoptosis in the testis is considered a crucial side effect of finasteride, a 5AR inhibitor used to treat BPH. Our results showed that AL treatment did not display such effects, while finasteride treatment resulted in loss of spermatogenic cells within the prostate. Overall, these results suggest AL as a potentially safe nature-derived therapeutic agent for BPH treatment.

## 1. Introduction

Benign prostatic hyperplasia (BPH) is a chronic disease commonly found especially in aging men [1]. It is reported that about 90% of males over an age of 80 suffer from BPH and lower urinary tract symptoms (LUTS) [2]. The main causes of BPH are pathologically increased proliferation of the smooth muscle in the prostate, leading to an abnormal enlargement of the prostate. Patients suffering from serious BPH display symptoms such as urinary intermittency, incomplete emptying, weak stream, staining, urgency, and nocturia [3].

The detailed mechanism of BPH pathogenesis is not fully revealed, but testosterone and dihydrotestosterone (DHT) are well known as inducers of BPH [4]. After the male sex hormone testosterone is produced, it is converted into DHT by 5*α*-reductase (5AR). DHT is considered a main factor of BPH pathogenesis, as it is expressed five times more than testosterone in BPH patients [4]. DHT has 2- to 10-fold higher binding affinity towards androgen receptor (AR) [5], a nuclear receptor which acts as a transcription factor after activation, and in result accelerates the proliferation of prostate cells. After conversion by 5AR, DHT combines with the AR and induces the expression of the growth factor, which leads to prostatic proliferation [6].

There are two mainly considered medical treatments for BPH. Alpha-blockers, which are inhibitors of *α*1-adrenergic receptors, relax the smooth muscle tone in the prostate and improve the urinal flow [7, 8]. However, the use of *α*-blockers is limited because of side effects such as headaches, hypotension, or ejaculation changes (absence of seminal emission, reduced ejaculation volume and force, etc.). Furthermore, the *α*-blocker itself cannot regulate the size of the prostate [9, 10]. Another class of medication is 5AR inhibitors, such as finasteride (Fi) and dutasteride. Suppression of 5AR by these inhibitors decreases the conversion of testosterone to DHT [11] and eventually leads to shrinking of prostate tissues. However, 5AR inhibitors can also cause side effects, such as loss of libido and erectile dysfunction [12]. Because of the side effects of existing medications, the need for alternative treatment is constantly growing [13].

Aconiti Lateralis Radix Preparata (AL), the processed daughter root of *Aconitum carmichaelii* Debx., of family Ranunculaceae, has been traditionally used as an essential herbal drug for cold sensation by stimulating heat generation in East Asia [14]. Studies have reported the anti-inflammatory, antitumor, and analgesic effects of AL [15–18], and moreover, its usage to treat colds, polyarthralgia, diarrhea, heart failure, beriberi, and edema are well known as well [19]. Nowadays, AL is clinically prescribed for diarrhea, syncope, joint pain, rheumatoid arthritis, and other inflammations [20].

The antiproliferative effect of AL on the prostate has been first reported by Dumbre et al. [21]. Dumbre's team showed that three ayurvedic plants including *Aconitum heterophyllum* suppressed prostate enlargement in testosterone-treated rats. However, this study only reports the antiproliferative effect lacking the related action mechanism. Therefore, we conducted a study to evaluate the effect of AL on BPH using testosterone propionate- (TP-) induced BPH rats and LNCaP prostate cancer cells.

## 2. Materials and Methods

### 2.1. Preparation of AL

The water extract of AL was obtained by boiling 100 g of dried roots of *Aconitum carmichaelii* Debx. in 1 L of distilled water at 100°C for 150 min based on the traditional extraction method of herbal prescriptions used in Korean medicine [22]. The solution was freeze-dried (yield 12.8%), filtered through a 0.22 *μ*m syringe filter, evaporated, and then stored at −20°C until usage.

### 2.2. Chemical Reagents

TP was purchased from Wako Pure Chemical Industries (Osaka, Japan), and Fi (≥97% pure) was purchased from Sigma-Aldrich Inc. (St. Louis, MO, USA). Antibody for AR was from Pierce Biotechnology (Rockford, IL, USA), and antibody for 5AR was from Abcam Inc. (Cambridge, MA, USA). Antibody for glyceraldehyde 3-phosphate dehydrogenase (GAPDH) was from Santa Cruz Biotechnology (Santa Cruz, CA, USA), and antibodies for BAX and Bcl-2 were purchased from Cell Signaling Technology (Danvers, MA, USA).

### 2.3. Animals

Twelve-week-old male Sprague Dawley (SD) rats (body weight 200–220 g) were purchased from the Dae-Han Experimental Animal Center (Dae-Han Biolink, Eumsung, Korea). The animals were all maintained in conditions in accordance with the regulation issued by the Institutional Review Board of Kyung Hee University (confirmation number KHUASP(SE)-P-034). The rats were housed in a pathogen-free room maintained at 23 ± 2°C under a 12 h light/dark cycle. Water and standard laboratory diet (CJ Feed Co., Ltd., Seoul, Korea) were provided ad libitum. BPH was induced as previously described [23], that is, by a 4-week pretreatment with daily subcutaneous injections of TP (5 mg/kg/day) in the inguinal region of rats. Fifty milliliters of the injection formula were prepared by dissolving 750 mg of TP in 5 ml of 100% ethanol and then mixing with 45 mL of corn oil. Each rat was injected with 100 *μ*L of this injection formula in the inguinal area. Normal control rats were injected with the same solvent without TP included. Then, the rats were divided into the following four groups (*n* = 5 per group) and were treated for 4 additional weeks: (a) a normal control (NC) group that received ethanol with corn oil, (b) a BPH group that received TP with corn oil, (c) a positive control group that received finasteride (Fi) (1 mg/kg/day) with TP (5 mg/kg/day), and (d) a group that received AL (20 mg/kg/day) with TP (5 mg/kg/day). AL and Fi were administrated via oral gavage. After the final treatment, animals were fasted overnight and euthanized using CO_2_, and the ventral region of prostate tissues was obtained as described previously [24]. The relative prostate weight index (prostate index (PI)) was calculated as the ratio of prostate weight (mg) to body weight (100 g). The prostate tissue was divided in half; one half was fixed in 10% formalin and embedded in paraffin for histomorphological assays, and the other was stored at −80°C for further assays.

### 2.4. Hematoxylin and Eosin (H&E) Staining

The prostate tissue section preparation and H&E staining were performed as described previously [25]. The slides were examined using the Olympus IX71 inverted phase microscope (Olympus Co., Tokyo, Japan). Epithelial thickness and lumen area fold were measured using ImageJ 1.47v software (National Institutes of Health, Bethesda, MD, USA).

### 2.5. Western Blotting Assay

Protein expression analysis was performed as previously reported [26]. In brief, homogenized prostate tissues or harvested LNCaP cells were lysed with ice-cold RIPA buffer, the insoluble materials were removed, and the proteins were separated by 8% sodium dodecyl sulfate-polyacrylamide gel electrophoresis and transferred onto polyvinylidenedifluoride (PVDF) membranes (Billerica, MA, USA). The membranes were then incubated with the primary antibody at 4°C overnight and subsequently incubated with HRP-conjugated AffiniPure Goat Anti-Rabbit IgG (Jackson ImmunoResearch Labs) or HRP-conjugated AffiniPure Goat Anti-Mouse IgG (Jackson ImmunoResearch Labs). The immunoblot intensity was quantified using ImageJ 1.47v software (National Institutes of Health).

### 2.6. Cell Culture

The human prostatic cancer cell line LNCaP was obtained from the Korean Cell Line Bank (Seoul, Republic of Korea). LNCaP cells were cultured in the Roswell Park Memorial Institute (RPMI) medium (Gibco, Big Cabin, OK, USA) supplemented with 100 mg/mL penicillin/streptomycin (HyClone, Logan, UT, USA) and 10% FBS (Sigma-Aldrich Inc.).

### 2.7. MTS Assay

LNCaP cells were seeded (2.5 × 10^4^ cells/well) and incubated in the RPMI medium plus 10% FBS for 24 h. Then, the cells were incubated in fresh media containing various concentrations of BBR for an additional 24 h. Cell viability was monitored using the cell proliferation MTS kit by the Promega Corporation as recommended by the manufacturer as previously described [27]. The absorbance was measured at 490 nm in a VersaMax microplate reader (Molecular Devices, Sunnyvale, CA, USA).

### 2.8. Ultrahigh-Performance Liquid Chromatography (UHPLC) Analysis

An Agilent 1290 Infinity II LC system (Santa Clara, CA, USA) with an Agilent 6550 iFunnel Q-TOF LC/MS system was used to perform high-resolution liquid chromatography-mass spectrometry (LC-MS) of AL according to the manufacturer's instructions by the Western Seoul Center of Korea Basic Science Institute. Data acquisition was performed using 6200 series TOF/6500 series Q-TOF B.06.01 (B6172 SP1) software (Agilent, Santa Clara, CA, USA). Specific conditions for LC-MS analysis are described in Table 1.

### 2.9. Statistical Analysis

The data were presented as mean ± standard deviation (SD). Statistical significance (*P* < 0.05) was analyzed by the Kruskal–Wallis H test followed by Bonferroni's method as a post hoc test using GraphPad Prism 5 for Windows (GraphPad Software, San Diego, CA, USA).

## 3. Results

### 3.1. AL Attenuates Prostatic Hyperplasia in TP-Induced BPH Rats

**Figure 1:**
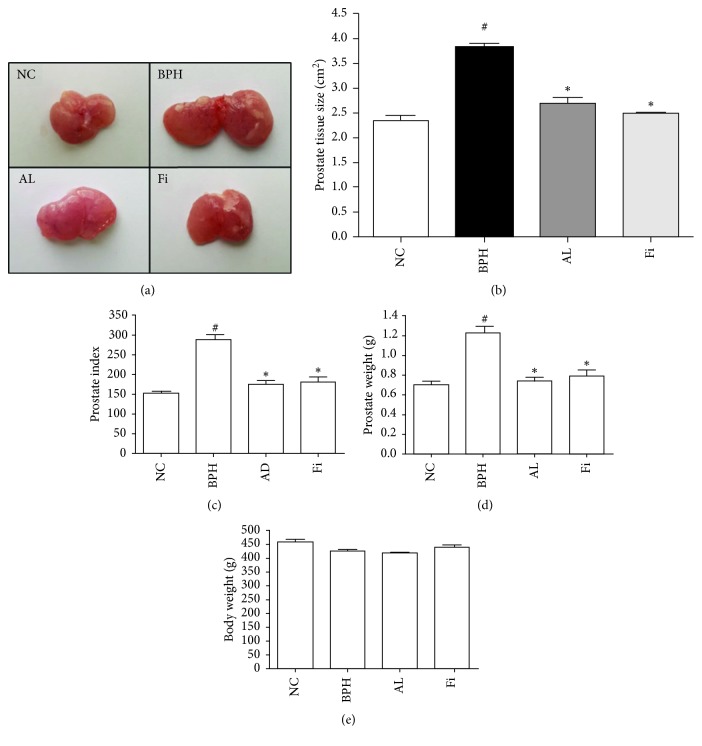
Effects of AL on prostate size and weight of TP-induced BPH rats. (a, b) Comparison of size of prostate tissues. (c) Prostate index value measured by dividing 100 g body weight by prostate tissue weight (mg). (d) Measurement of prostate weight of rats. (e) Measurement of body weight of rats. Values are expressed as mean ± SD. ^#^ *P* < 0.05 when compared to NC; ^*∗*^ *P* < 0.05 when compared to BPH. NC, normal control group; BPH, TP-induced BPH group; AL, AL-treated BPH group; Fi, finasteride-treated BPH group.

Changes in prostate tissue size, prostate weight, and prostate index are shown in Figure 1. Rats treated with TP showed an increase in prostate weight and PI by 1.74-fold and 1.88-fold compared to the normal control. AL treatment significantly decreased the prostate weight by 70% and PI by 74%. This effect was relatively higher than that in the Fi group, rats of which showed decreased prostate weight by 62% and PI by 70%. Body weight of rats did not show any difference among all groups (Figure 1(e)).

### 3.2. AL Restores Histological Changes of Prostate Tissues in TP-Induced BPH Rats

**Figure 2:**
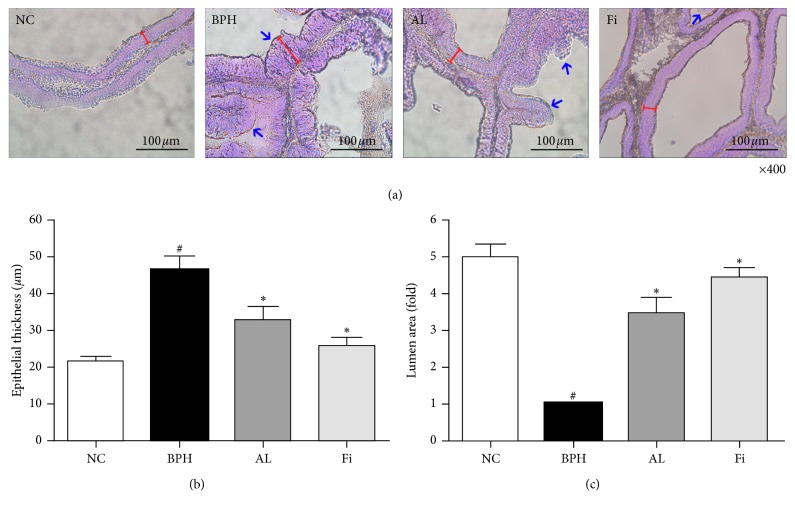
Effects of AL on histological changes in prostate tissues of TP-induced BPH rats. (a) Representative photomicrographs of H&E-stained prostate tissues (magnification ×400). Measurement of the (b) epithelial thickness and (c) relative lumen area of the prostate tissues using ImageJ software. Values are expressed as mean ± SD of ten or more separate measurements. ^#^ *P* < 0.05 when compared to NC; ^*∗*^ *P* < 0.05 when compared to BPH. NC, normal control group; BPH, TP-induced BPH group; AL, AL-treated BPH group; Fi, finasteride-treated BPH group.

As shown in Figure 2(a), histology of the prostate was assessed by H&E staining. TP administration caused various histological changes in the prostate. The epithelium of the prostate gland with BPH displayed signs of proliferation such as increased epithelial thickness, overformed acinus area, and decreased lumen area. However, AL-treated rats and Fi-treated rats showed recovered histology similar to NC rats, with less pathological tissue structures (Figures 2(b) and 2(c)).

### 3.3. AL Regulates 5AR-AR Axis in TP-Induced BPH Rats and LNCaP Cells

**Figure 3:**
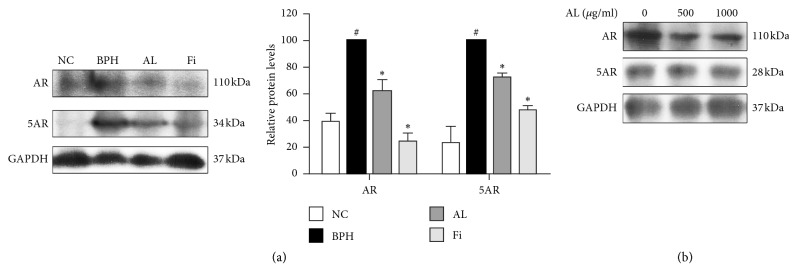
Effects of AL on expressions of 5AR and AR in prostate tissues of TP-induced BPH rats and LNCaP prostate cancer cells. Western blot analysis of the protein expressions of 5AR and AR from (a) prostate tissues of TP-induced BPH rats and (b) LNCaP prostate cancer cells. Protein expression values were normalized to GAPDH. Values are expressed as mean ± SD of three separate measurements. ^#^ *P* < 0.05 when compared to NC; ^*∗*^ *P* < 0.05 when compared to BPH. NC, normal control group; BPH, TP-induced BPH group; AL, AL-treated BPH group; Fi, finasteride-treated BPH group.

The factors related to prostatic hyperplasia were examined by western blot assays using antibodies against 5AR and AR. These factors were increased in the TP-treated BPH group compared to those in the NC group. However, as shown in Figure 3(a), 5AR and AR were significantly decreased by AL treatment when compared to the TP-treated BPH group (0.73-fold and 0.63-fold, respectively). This was further confirmed in vitro, as AL treatment inhibited the expression of 5AR and AR in LNCaP prostate cells (Figure 3(b)), even though AL treatment up to 1000 *μ*g/mL did not affect cell viability of LNCaP cells (Supplementary Figure S1).

### 3.4. AL Elevates BAX/Bcl-2 Ratio in TP-Induced BPH Rats and LNCaP Cells

**Figure 4:**
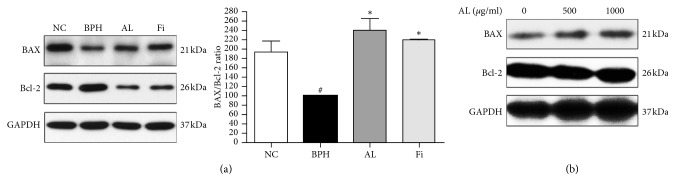
Effects of AL on BAX/Bcl‐2 ratio in prostate tissues of TP-induced BPH rats and LNCaP prostate cancer cells. Western blotanalysis of the protein expressions of BAX and Bcl‐2 from (a) prostate tissues of TP‐induced BPH rats and (b) LNCaP prostate cancer cells.Values are expressed as mean ± SD of three separate measurements. ^#^ *P* < 0.05 when compared to NC; ^∗^ *P* < 0.05 when compared to BPH.NC, normal control group; BPH, TP‐induced BPH group; AL, AL‐treated BPH group; Fi, finasteride-treated BPH group.

We then evaluated the effect of AL on the ratio of BAX and Bcl-2. BAX/Bcl-2 ratio is considered an indicator of cell apoptosis, and Saker et al. have shown this ratio is important in the pathogenesis of both BPH and prostate cancer [28]. The ratio of BAX and Bcl-2 was decreased in prostate tissues of TP-induced rats, and this was restored to a level similar to that in the NC group by AL and Fi treatment (Figure 4(a)). Similar effects were observed in AL-treated LNCaP cells as well (Figure 4(b)), suggesting an apoptotic induction by AL in prostate cells.

### 3.5. AL Treatment Does Not Cause Pathological Changes in Testis

**Figure 5:**
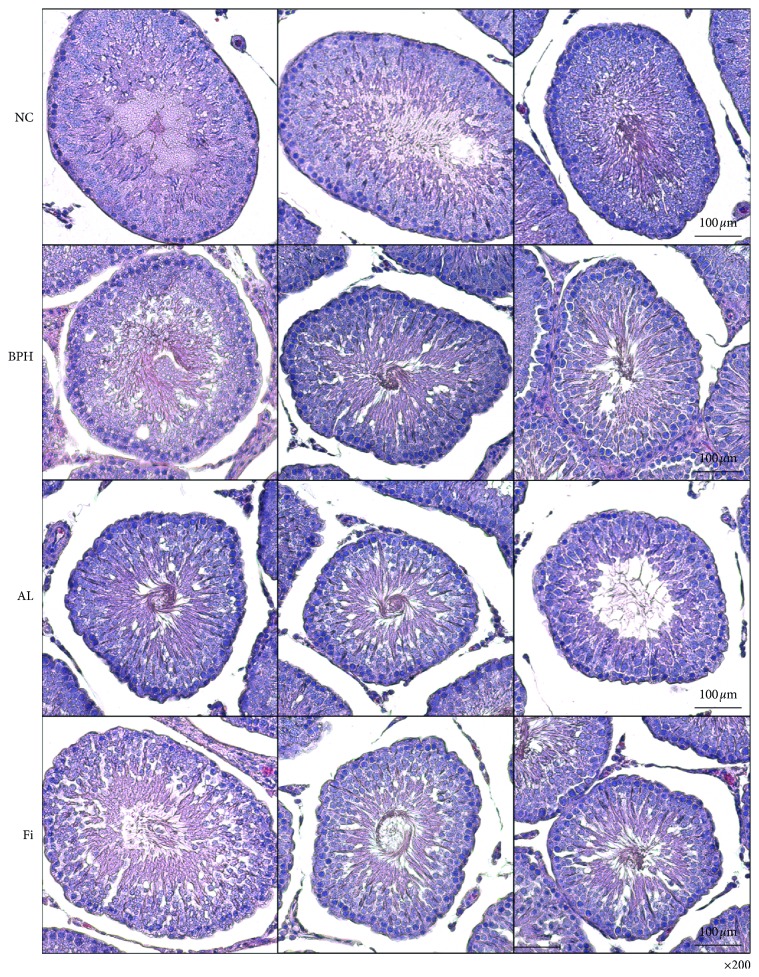
Effects of AL on histological changes in testis tissues of TP-induced BPH rats. Representative photomicrographs of H&E-stained testis tissues (magnification ×200) are shown. NC, normal control group; BPH, TP-induced BPH group; AL, AL-treated BPH group; Fi, finasteride-treated BPH group.

One of the fatal side effects of 5AR inhibitors such as Fi is apoptotic enhancement in the testis [29, 30]. Based on these studies, we compared the histology of the testis of Fi- and AL-treated rats. As shown in Figure 5, the testicular tissue showed a proper arrangement of germinal cells in the NC and BPH groups, with no histopathologic lesions. However, in the Fi group, lower spermatogenic cell density was observed, which was improved in AL-treated rats. These results show that AL does not display toxicity in the testis and does not impact spermatogenesis.

### 3.6. Chromatographic Analysis of AL

**Figure 6:**
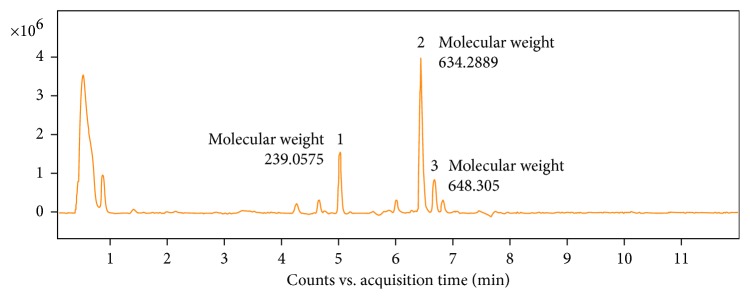
UHPLC analysis of AL: the representative chromatogram of AL and identification of its components.

We used the UHPLC method to analyze components of AL. As a result, we could identify three components with molecular weights of 239.0575, 634.2889, and 648.305 (Figure 6). Further information on the spectrum peaks is presented in Table 2.

## 4. Discussion

Prostate diseases can be categorized into three groups: prostatitis, BPH, and prostate cancer. Among the three diseases, BPH is the most common disease in men [31]. Prostate tissue consists of four areas: the central zone, peripheral zone, transition zone, and anterior fibromuscular stroma. BPH associates with an overproliferated transition zone and stiffness of the prostatic smooth muscle tone. These abnormal changes in the size and muscle tone cause compression on the urethra, thus leading to LUTS, a common class of symptoms in BPH [32]. The main reason which makes BPH a serious health issue is epidemiology: around 50% of males over 50 and 90% over 80 suffer from the symptoms of this chronic illness [2].

The pathological process of BPH associates with several molecular mechanisms. As described above, the 5AR-AR axis is considered the most crucial pathway of BPH. Besides 5AR and AR, the balance between cell proliferation and apoptosis is also closely related [33]. Apoptosis starts by two different programmed mechanisms. The extrinsic apoptosis is initiated by activation of Fas and tumor necrosis factor receptor 1, followed by cleavage of caspase-8 [34], while the intrinsic pathway begins by release of cytochrome c from the mitochondria and a subsequent activation of caspase-9 [35], and both pathways meet at the final few stages including cleavage of caspase-3, the effector caspase [36]. The intrinsic apoptosis is regulated by Bcl-2, a mitochondrial outer membrane-located protein which inhibits release of the proapoptotic factor cytochrome c [37]. The BAX gene was the first to be identified as a proapoptotic member among the Bcl-2 family [38]. BAX protein forms a heterodimer with Bcl-2 and then activates the apoptosis signal. Thus, the ratio of BAX and Bcl-2 determines the apoptotic sensitivity [39]. Under the normal condition, prostate tissues express relatively low levels of Bcl-2 and caspase-3, associated with a low level of apoptosis [40].

AL is the dried root of *Aconitum carmichaelii* Debx., a flowering plant of the genus *Aconitum*. As a native plant of East Asia, AL in traditional medicine has been used for centuries to treat extremely cold symptoms in China and Korea [41]. Although AL is extensively used as a potent herb in traditional Korean medicine, its toxicity requires careful preparation. Aconitine and related alkaloids are toxins which can impact the sympathetic-parasympathetic nerve systems and cardiovascular system [42]; however by heat-processing, they can be changed into rather safer alkaloids [43]. In this study, we investigated the possible use of AL as a novel herbal medication for BPH treatment.

The TP-induced BPH animal model [44] has been widely used for BPH studies because this model displays similar histologic characteristics and pathological abnormalities to humans as described by McNeal [45]. AL treatment suppressed the TP-induced enlargement of the prostate by 70% in prostate tissue weight and 74% in PI. The inhibition rate on both factors was higher than that of Fi treatment (62% in prostate tissue weight and 70% in PI). The pathohistological changes such as epithelial thickness and lumen area were recovered by AL as well. TP injection resulted in an increase of epithelial thickness and decrease of lumen area, associated with the increase of acinus numbers. AL-treated rats showed decreased thickness of the epithelium and increased lumen area while reducing the number of acini.

In addition, the two main pathways responsible for abnormal proliferation of the prostate, androgen-related pathway and apoptosis pathway, were regulated by AL treatment. As thoroughly described, the androgen-related pathway is a crucial pathogenic pathway of BPH. When testosterone is converted into the highly affinitive hormone DHT [5] by 5AR, it binds to the nuclear receptor AR and results in increased proliferation by the transcriptional activity of AR [6]. Studies including our previous ones showed improvement of BPH by suppressing these two factors [23–27]. Our current results also demonstrated that AL can reduce the expressions of 5AR and AR in both TP-induced BPH rats and LNCaP prostate cancer cells, suggesting the inhibitory effect of AL on the androgen pathway during the pathogenesis of BPH.

The balance between proliferation and apoptosis, which can be identified by BAX/Bcl-2 ratio, is also closely related to BPH [33]. While normal prostate shows a relatively low level of apoptosis, in BPH situations, imbalance between apoptosis and proliferation is displayed either by decreased apoptotic signals [40] or by increased proliferative factors such as proliferating cell nuclear antigen (PCNA) [46] and Ki67 [47]. In this study, AL treatment showed the restoration of BAX/Bcl-2 ratio which was significantly decreased by TP. In LNCaP cells, AL treatment increased the ratio of BAX and Bcl-2, indicating induced apoptosis in this prostate cancer cell line.

In addition to the suppressive effect of AL on 5AR and AR, AL treatment also significantly increased the ratio of BAX and Bcl-2, indicating upregulated apoptosis. Corresponding to previous reports [40], prostate tissues of NC rats showed relatively lower levels of Bcl-2 and higher levels of BAX, which were pathologically changed by TP injection. AL treatment restored the BAX/Bcl-2 ratio similar to that of NC rats. Similar results were observed in LNCaP prostate cancer cells, and AL treatment dose-dependently increased the ratio of BAX and Bcl-2.

5AR inhibitors such as Fi reduce the size of the prostate gland by inhibiting the activity of 5AR. However, sex-related side effects are frequently reported, including pathological apoptosis in the testis [29, 30]. Our results also demonstrated decreased cell density caused by Fi on the testis tissue, but AL-treated rats did not show any abnormal histological changes in the testis.

Hit identification is the basic level of small molecule discovery, and therefore, it is necessary to identify effective compounds to value the effects of natural products. Our LC-MS analysis identified three constituents which may possibly be responsible for the beneficial effect of AL on BPH. Although the molecular weight of identified compounds did not match exactly with that of previously known constituents of AL, based on previous reports [48, 49], there is a possibility that component 1 (molecular weight: 239.0575) could be higenamine (C_16_H_17_NO_3_; molecular weight: 271.316), component 2 (molecular weight: 634.2889) could be hypaconitine (C_33_H_45_NO_10_; molecular weight: 615.72) or mesaconitine (C_33_H_45_NO_11_; molecular weight: 631.719), and component 3 (molecular weight: 648.305) could be aconitine (C_34_H_47_NO_11_; molecular weight: 645.746). However, to clarify, further studies should be carried out with different fractions and/or identified constituents to confirm which is the active compound responsible for the effects of AL.

## 5. Conclusions

In conclusion, we confirmed increased levels of 5AR and AR and a decreased ratio of BAX and Bcl-2 as well as the changes of the histological structure in BPH rats. These changes were reversed by AL treatment, and in addition, AL-treated LNCaP cells also showed similar results. Furthermore, AL treatment did not induce any abnormal changes in the histology of the testis as Fi treatment did. These results suggest AL as a potentially safe nature-derived therapeutic agent for BPH treatment.

## Figures and Tables

**Table 1:** Analysis conditions for LC-MS.

LC-MS condition			
Component	Agilent 1290 Infinity II UHPLC		
	G6650A Q-TOF MS (Agilent)		
Ion source	Dual AJS ESI		
Polarity (kV)	Positive mode (4.0)		
	Negative mode (3.5)		
Mass range (*m*/*z*)	20–1700		
Reference masses (*m*/*z*)	Positive mode with 121.0509 and 922.0098		
	Negative mode with 112.9856 and 966.0007		
LC column	Agilent Eclipse Plus C18 RRHD column (50 mm × 2.1 mm, 1.8 *μ*m)		
LC flow rate	0.300 mL/min		
Injection volume	1.00 *μ*l		
Column temperature	30°C		
Solvent composition	A: 0.1% formic acid in water		
	B: 0.1% formic acid in ACN		
Mobile phase	Time (min)	A (%)	B (%)
	0.0	95	5
	3.0	95	5
	13.0	10	90
	15.0	10	90
	17.0	95	5
	20.0	95	5

**Table 2:** MS spectrum peak list.

No.	*m*/*z*	Abundance	Ion	Expected formula	Score
1	239.0575	764608.06	(M-H)-	C_6_H_14_N_3_O_5_S	88.81
2	634.2889	1565763	(M-H)-	C_20_H_29_N_25_O	96.31
3	648.305	331263.47	(M-H)-	C_20_H_45_N_10_O_14_	97.24

## Data Availability

All raw data supporting the results of this study are available from the corresponding author upon request.

## Supplementary Materials

Supplementary Materials Supplementary Figure S1: effects of AL on cell viability in LNCaP cells. The MTS assay was performed in order to measure the effect of AL on cell viability in LNCaP cells.
