# Supplementary figures and images for: Aconiti Lateralis Radix Preparata, the Dried Root of Aconitum carmichaelii Debx., Improves Benign Prostatic Hyperplasia via Suppressing 5-Alpha Reductase and Inducing Prostate Cell Apoptosis
Source: Evid Based Complement Alternat Med. 2019 Jul 31;2019:6369132. doi: 10.1155/2019/6369132 (PMC6701400; doi:10.1155/2019/6369132)

509    **Supplementary Figure S1.**

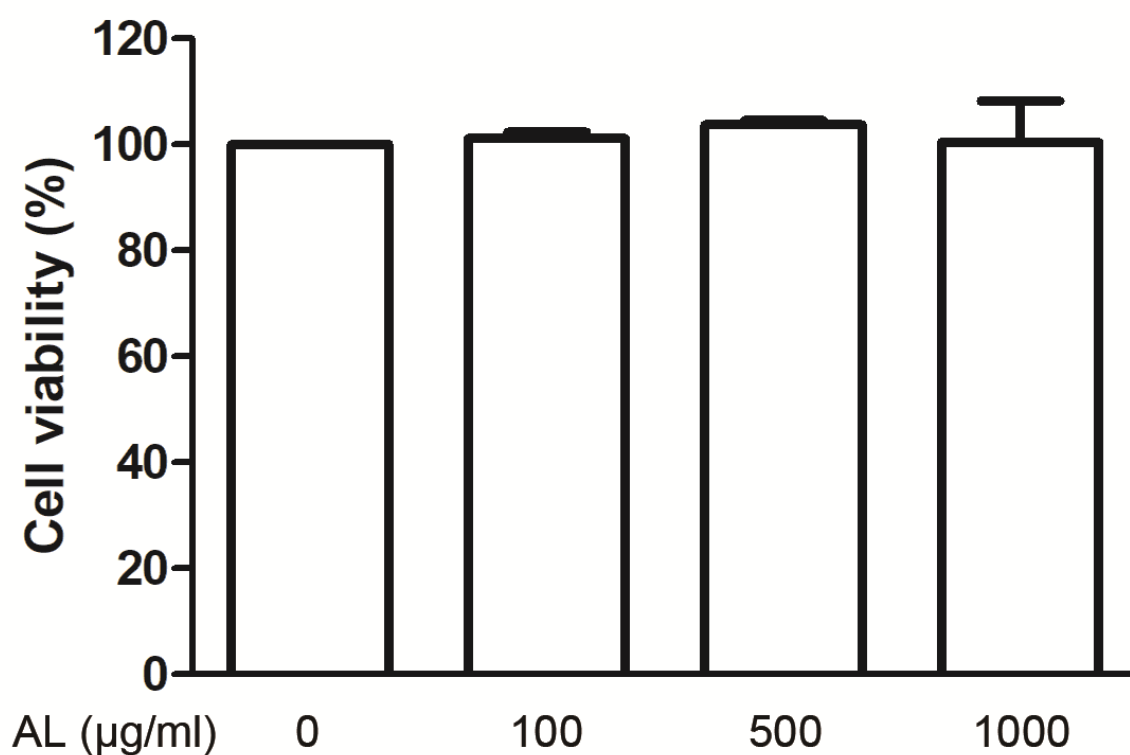

510

Supplement: Supplementary Materials — Supplementary Figure S1: effects of AL on cell viability in LNCaP cells. The MTS assay was performed in order to measure the effect of AL on cell viability in LNCaP cells. [file 6369132.f1.pdf]
